# Progress in Gynæcology

**Published:** 1904-02-13

**Authors:** 


### PROGRESS IN GYNÆCOLOGY.

(*Concluded from page* 338.)

Ectopic Gestation.—Ward Cousins,[7] in an instructive paper on this subject, reviews the conditions that favour the advance of the pregnancy to full term. He considers them under the following headings:—(1) The early and continuous mobility of the ovum. Mobility of the gestation sac is generally well marked during the early months, and the continued development of the fœtus depends to a large extent on this mobility being maintained; if the sac develops in such a position that its mobility is hindered, the chances of continued growth and vitality are greatly reduced; (2) gradual expansion of the gestation sac. In cases that advance to full term, the wall of the gestation sac appears to undergo a slow process of stretching without any marked laceration of its fibrous structure. If a slight rupture should take place, it is with few exceptions extra-peritoneal, and the opening is soon securely plugged without any serious hæmorrhage or disturbance of the placental circulation; (3) early ascent of the fœtus towards the abdominal cavity. It is almost impossible for a case in which the gestation sac becomes locked in the pelvis, and stopped in its ascent towards the abdominal cavity to proceed beyond about the fifth month; (4) the position of the placenta. The course of an ectopic gestation is greatly influenced by the position of the placenta, and Ward Cousins recognises three positions in which it may be placed: (*a*) it may be situated at the bottom of the sac; (*b*) it may be uppermost and attached to the upper part of the wall of the sac; or (*c*) it may be laterally incorporated with the sac, and be elongated and displaced upwards for many inches into the abdominal cavity. Of these the last is the most favourable site for maintaining the vitality of the child. An interesting case of hæmatosalpinx due to tubal pregnancy and complicated by torsion of the pedicle is recorded by F. J. McCann.[8] On examination a smooth rounded swelling was detected occupying the right posterior quarter of the pelvis. The patient had suffered from several severe attacks of pain, which were distinctly abdominal rather than pelvic. On opening the abdomen the swelling was found to be a dilated tube containing blood clot, chorionic villi, and fluid blood, the pedicle being twisted three times. The tumour was removed and the patient made a good recovery.

Gonorrhœa in the Female.—W. E. Fitch[9] maintains that gonorrhœal inflammation in the female rarely presents symptoms physically analogous to those observed in the male. Vaginitis of venereal origin is exceptional, and urethritis is rare. The inherent toughness of the vagina allows it to act as a vessel where the toxic material may be elaborated, and infect the endometrium. Fitch believes that the majority of pelvic diseases in women are due to gonorrhœa, and that the gonococci do not lose their virulence even after laying dormant for years in the genital tract, but may spring into new life under the favouring influence of the pregnant state, or a curettage. As regards treatment he lays stress on the great importance of rest, especially during the menstrual period. Vaginal injections of potassium permanganate 1—2,000 and 1—1,000 are very useful, when the acute stage has subsided. Urethritis and vesical complications require balsams and alkalies, or anodynes and sedatives may be indicated. If there is much pain and a frequent desire to pass water, urinary antiseptics and vegetable diuretics will give relief. R. O. Kevin[10] says that the patient should be kept in bed during the acute stage, and no local treatment undertaken. A powder containing salol, pot. bicarb. and sodæ brom.

given every two hours with much water will lessen the ardor urinæ. When the very acute stage has subsided he recommends that the urethra should be injected with about half an ounce of a 5—10 per cent. solution of argyrol. Should infection persist urotropin should be given internally, with endoscopic application of a solution of argent. nit. to the neck of the bladder.

**The Etiology of Tubal Pregnancy.**—Micholitsch[11] has examined the specimens from a large number of cases, and says that in none of them did he find the ovum implanted in a normal tube. The abnormalities were either congenital or acquired. In every instance the deformity caused some mechanical obstruction to the passage of the ovum, folds of mucosa, secondary lumina, or pockets. He believes the presence of these secondary lumina or pockets in the tube to be the chief cause of tubal pregnancy. In the cases in which the opposite tube also could be examined, he found similar changes, showing that the tubal lesions were not secondary to the development of the ovum, but preceded its implantation. Hahn[12] says that he believes that gonorrhœa is undoubtedly the most frequent cause of extra-uterine pregnancy. The affection is most common, he says, where gonorrhœa is most common, that is to say, in the great cities. He affirms that the number of cases of ectopic gestation now met with is greater than formerly, and this increase may be accounted for in two ways, (1) because gonorrhœal infection has spread more widely than ever, and (2) because increased experience has so improved the diagnosis of ectopic pregnancy that very few cases are now overlooked.

**Fibromyoma of Vagina.**—Potel,[13] from an analysis of 160 reported cases, says that the tumours may be either pedunculated or sessile, the former being the more common. In the sessile variety the tumour generally possesses a capsule from which it can be enucleated, but sometimes the growth is intimately connected with the surrounding tissues. The tumours vary much in size, sometimes they are quite small, at others they may completely fill the vagina, and even cause an abdominal swelling. The co-existence of a fibrous tumour of the uterus and vagina is rare. The tumour may arise from any part of the vagina, but the anterior wall is most frequently involved. The growth is generally composed of fibrous and muscular tissue in varying proportion.

[7] Brit Med. Jour., July 25, 1903. [8] Lancet, May 9, 1903. [9] Amer. Jour. of Obstetrics and Diseases of Women, Oct., 1903. [10] New York Med. Jour., June 20, 1903. [11] Amer. Jour. of Obstetrics and Diseases of Women, Sept., 1903. [12] Brit. Med. Jour., June 20, 1903. [13] Jour. of Obstetrics and Gynæcology of the British Empire, Aug., 1903.

